# A Review of Self-Poisoning Admissions in a Five-Bed Intensive Care Unit in Scotland

**DOI:** 10.1186/2197-425X-3-S1-A497

**Published:** 2015-10-01

**Authors:** G Warnock, L Young

**Affiliations:** Victoria Infirmary/ NHS Greater Glasgow & Clyde, Intensive Care Department, Glasgow, United Kingdom

## Introduction

Deliberate self-poisoning is a common presentation to hospitals, with 13,268 presentations to Scottish hospitals in 2004 accounting for 1.11% of total admissions [[1]]. It accounts for up to 4% of ICU admissions, with the majority being under forty years old and predominantly male [[2]]. in a recent study of 481 patients, the average length of stay was 0.7 days, and hospital mortality was 4% (n = 20) [[2]].

## Objectives

To assess the epidemiology and outcomes of self-poisoning admissions to our local intensive care unit, and to quantify which substances are currently most prevalent.

## Methods

A WardWatcher^TM^search was performed retrospectively for all admissions with the primary unit diagnosis equal to 'self-poisoning'. the search returned 330 records (approximately 4.8% of all admissions). We excluded any records that were alcohol poisoning only, had no record of what substances were taken, or were missing APACHE data (n = 156).

## Results

A total of 174 records were included in the analysis. the mean age was 37, and there was a slight propensity for male gender (F:M = 1:1.15). the mean length of stay was 1.8 days, with 39% (n = 68) staying less than one day. the mean APACHE mortality prediction was 15.3%. Actual unit mortality was 2.3% (n = 4), with a standardised mortality ratio (SMR) of 0.15. the most common presentation was that of a mixed overdose in 46.6% (n = 81); the commonest substances taken included anti-depressants in 42.5% (n = 74), paracetamol-containing drugs in 14.9% (n = 26), and benzodiazepines in 21.3% (n = 37). Alcohol was also involved as part of the mixed overdose in 25.2% (n = 44).

## Conclusions

Our review shows a similar demographic for self-poisoning admissions - patients were under forty and there was a slight propensity for male gender. the actual mortality in our dataset was lower than that reported elsewhere, and significantly the SMR was less than one. the most prevalent substance taken in our review was anti-depressants.
